# Correlation and influencing factors analysis of colorectal polyps with *Helicobacter pylori* Infection and p-S6K1 expression

**DOI:** 10.1186/s12879-023-08791-y

**Published:** 2023-11-14

**Authors:** Zeming Jia, Xiaoping Wan

**Affiliations:** Department of General Surgery, Xiangya Hospital, Central South University, No. 87 Xiangya Road, 410008 Changsha, Hunan P R China

**Keywords:** Colorectal polyps, *Helicobacter pylori* Infection, Correlation, p-S6K1, Clinical significance

## Abstract

**Objective:**

To investigate the correlation between colorectal polyps (CRP) and *Helicobacter pylori (H. pylori)* infection, and the correlation between CRP and the expression of phosphorylated ribosomal protein S6 kinase (p-S6K1). Besides, its related influencing factors were determined in the present study.

**Methods:**

A total of 191 subjects who underwent colonoscopy in our hospital from January 2020 to February 2022 were selected for this study. Among them, 141 patients were diagnosed with CRP, and the other 50 subjects were no significant colorectal abnormalities. 141 CRP patients were divided into *H. pylori*-positive group (n = 89) and *H. pylori*-negative group (n = 52) according to the results of the *H. pylori* test. The expression of p-S6K1 in CRP tissue was detected. The relationship between the p-S6K1 expression and the clinicopathological characteristics of CRP patients was analyzed. The logistic analysis of factors influencing the occurrence of CRP was performed.

**Results:**

There were significant differences in pathological type, site of disease, the number and size of polyps between the *H. pylori* negative group and the *H. pylori* positive group (*P* < 0.001, *P* = 0.037, *P* = 0.042 and *P* = 0.039). The percentage of the p-S6K1 positive expression in polyp tissues was higher than that in normal tissue and parapolyp tissues (*P* < 0.001). The p-S6K1 negative group showed significant difference in the number and pathological type of polyps and the presence or absence of a pedicle as compared with the p-S6K1 positive group (*P* = 0.006, *P* < 0.001 and *P* = 0.012). Logistic multifactor analysis showed that BMI, *H. pylori* infection, smoking history, ApoB, Lp(a) and the p-S6K1 positive expression were all risk factors for the development of CRP (*P* = 0.025, *P* = 0.020, *P* = 0.010, *P* = 0.005, *P* = 0.043 and *P* < 0.001).

**Conclusion:**

*H. pylori* infection was closely related to the pathological type, location, and the number and size of CRP. p-S6K1 was highly expressed in CRP, and was positively related to the number, the pathological type and pedicle of polyps. *H. pylori* infection and the positive p-S6K1 expression were independent risk factors for CRP. By exploring the association between *H. pylori* infection as well as p-S6K1 and CRP, it is hoped that it will help to formulate a more rigorous colorectal cancer screening program for *H. pylori*-positive individuals, and at the same time find a new direction for the prevention of CRP and colorectal cancer, and provide some help for future research.

## Introduction

Colorectal polyps (CRP) refer to protrusions that extend from the usually flat mucosa of the colon to the lumen, which can be classified into tumor polyps and non-tumor polyps based on their histological characteristics and susceptibility to malignancy [1]. Among them, adenomatous polyps are considered as precancerous lesions of colorectal cancer (CRC) that is one of the most common malignant tumors worldwide [2]. Therefore, discovering the risk factors for CRP plays is important to reduce or avoid the occurrence of CRC. *Helicobacter pylori* (*H. pylori*) is a common gram-negative bacterium. *H. pylori* infection releases a large number of inflammatory factors, which can induce chronic gastritis and gastric atrophy, as well as increase the probability of transformation into cancer [3]. Thus, *H. pylori* is considered to be the first biological carcinogen for the induction of cancer. Because *H. pylori* infection induces a long process of cancer, it may be related to the destruction of certain genes and protein factors by *H. pylori*, which finally results in an irreversible damage to the gastric mucosa [4].

Phosphoribosomal S6 Kinase 1 (P-S6K1) is a key downstream effector of the mammalian rapamycin target (mTOR) pathway, which is considered an indirect marker of mTOR activity. mTOR is a protein kinase, whose abnormal activation can significantly promote the proliferation and migration of CRC cells. The mTORC1 complex is related to the body’s inflammatory response, innate immune response and bacterial infection, whose high expression is correlated with gastric mucosal lesions and the occurrence and development of gastric cancer [5]. Abnormally high expression of S6K1 protein in CRC mucosal tissues indicate that S6K1 may have an important effect on accelerating the formation of CRC [6]. The expression of S6K1 protein in CRC mucosal tissues is abnormally high, which was related to the clinical stage and tissue differentiation of patients, suggesting that S6K1 protein plays an important role in the malignant transformation of CRC epithelial cells [7]. However, it is still unclear whether the association between *H. pylori* infection and CRP exists, and the expression of p-S6K1 in CRP tissue is not yet known.

The study has confirmed that patients with *H. pylori* infection have a significantly increased risk of colorectal cancer compared with patients without *H. pylori* [8]. Therefore, it is important to discover the factors that influence the cancer caused by *H. pylori* infection. In this study, the expression level of p-S6K1 in normal colon tissues and CRP tissues was mainly examined, and its clinical significance was discussed, so as to further understand the relationship between *H. pylori* infection and the p-S6K1 expression level, and explore the possible pathogenesis and process of the CRP formation caused by *H. pylori* infection, ultimately providing a reference for early clinical diagnosis and treatment of CRP.

## Materials and methods

### General materials

**Fig. 1:**
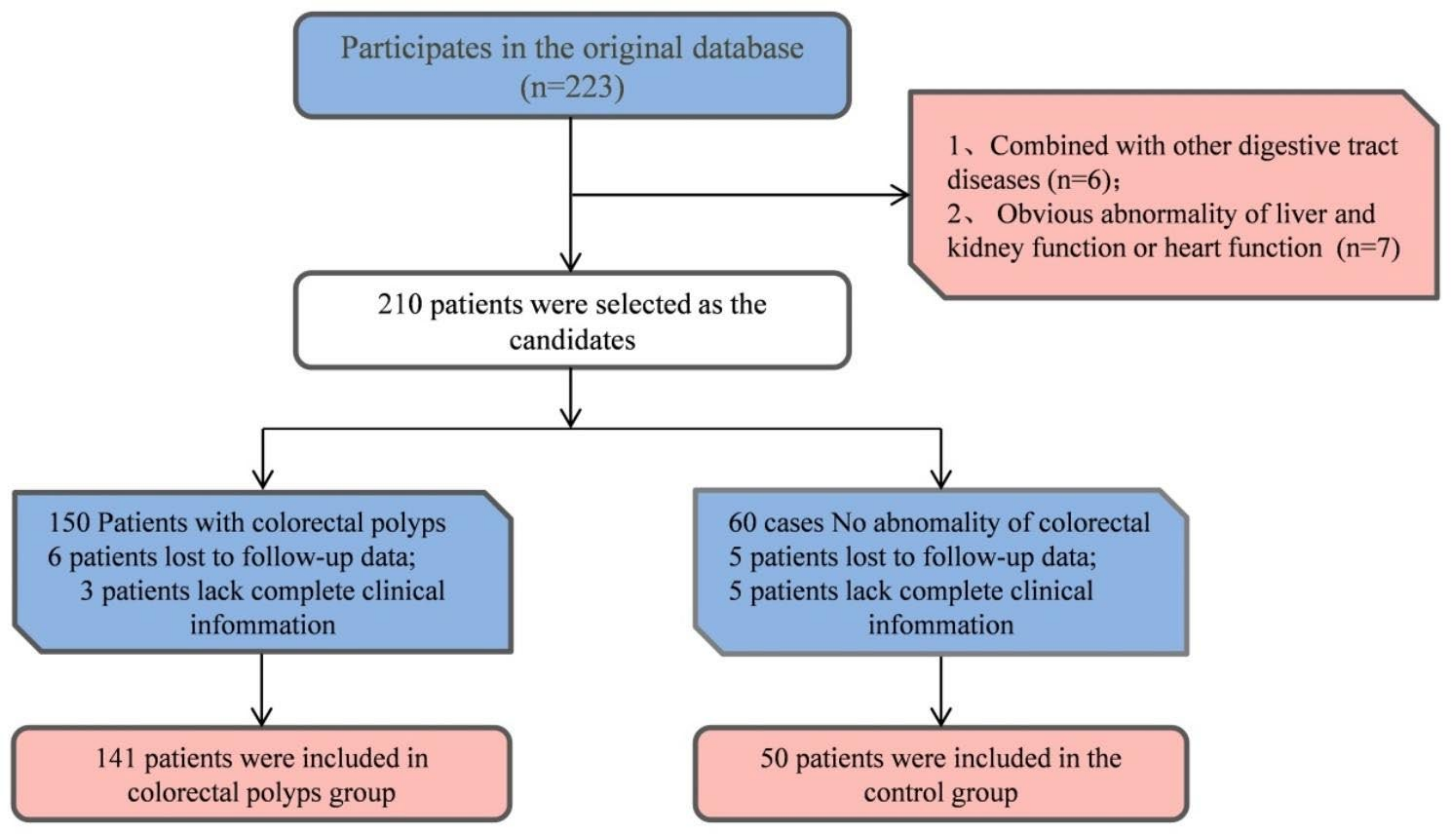
The general data selection process for 191 patients

Before the initiation of the study, the PASS software was used to calculate the sample size, according to the formula n = 2*[(α + β)σ/δ]^2. δ was the required degree of differentiation; σ was the population standard deviation or its estimated value s; α (0.05) and β (0.10) were the u values corresponding to the α and β, which could be found by the t-boundary value table and the degrees of freedomυ=∞-line. α was divided into one-sided and double-sided, in which only one-sided values were β. Considering 20% shedding rate, the sample size was estimated [9] and the case data were collected through the case room. The clinical data of 191 patients who underwent colonoscopy in our hospital from January 2020 to February 2022 were selected for the study. Among them, 141 patients were diagnosed with CRP. Besides, the other 50 subjects with no significant colorectal abnormalities were taken as the control. The CRP patients were further divided into *H. pylori*-positive group (n = 89) and *H. pylori*-negative group (n = 52) according to the results of the 13 C-urea breath test. Inclusion criteria: (1) CRP patients met relevant diagnostic criteria [1] and were diagnosed by the colonoscopy and pathological tissue examination; (2) The patient did not receive relevant treatment before the study; (3) All patients underwent 13 C urea breath test testing. Exclusion criteria: (1) Patients with concomitant digestive system diseases; (2) Patients with incomplete clinical and pathological data; (3) Patients with concomitant mental disorders and low research cooperation; (4) Patients with significant abnormalities in liver and kidney function or cardiac function; (5) Pregnant or lactating patients. The general data selection process for 191 patients was shown in Fig. 1.

### Immunohistochemical (IHC) assay [10]

CRP tissues, polyp adjacent tissues, and normal tissues were taken from surgery or colonoscopy pathological examination, with at least 3 cm away from the polyp tissues as the adjacent tissue and at least 5 cm away from the normal tissues. Among them, surgical patients met the following indications: The lesions of adenomatous polyps, tubular adenoma polyps, villous adenoma polyps, and tubular villous mixed adenoma polyps ≥ 6 mm, microscopic polypoid adenomas ≤ 5 mm, flat and depressed tumor lesions, even if ≤ 5 mm should be endoscopically removed. All tissues were made into 4 μm thick paraffin sections. After being dewaxed with xylene, dehydrated with gradient ethanol, and washed with distilled water, the slices were incubated with a 3% hydrogen peroxide solution at room temperature for 15 min. The slices were put in citrate buffer for antigen repair for 10 min, and then incubated at 4℃ for 12 h with p-S6K1 antibody. The slices were incubated with horseradish peroxidase labeled secondary antibodies. The next day, they were taken out and soaked in phosphate solution three times, each time for 3 min. DAB color development for 3–5 min, followed by hematoxylin re-staining, rinsing with tap water, dehydration, transparency, and sealing. 4 fields of view were selected under each high-power microscope. The expression level of p-S6K1 in CRP tissues, adjacent polyp tissues, and normal tissues were valued using IHC. The staining results of tissue slices were judged by at least three experienced doctors. The expression of p-S6K1 was evaluated by the immunohistochemistry score, that is, staining intensity × positive cell rate, in which 0 point was negative, ≤ 4 points were weak positive, and > 4 points were strong positive. Weak positive and strong positive were regarded as the positive expression. The scoring method was as follows: (1) dyeing intensity: the rating of uncolored dyeing intensity was 0 points, light yellow was 1 point, brown yellow was 2 points, and tan was 3 points; (2) Cell positive rate: Five fields of view at 400 × were taken to calculate the percentage of positive cells in the field of view. The calculation for the percentage of positive cells was relative to the total number of cells in the field of view by taking its mean. < 5% scored 0 points, 5% ≤ cell positive rate < 25% scored 1 point, 25% ≤ cell positive rate < 50% scored 2 points, cell positive rate ≥ 50% scored 3 points.

### Outcome measures

The pathological type (non-adenomatous, adenomatous), site of onset (left, right, left + right), the number of polyps (1, > 1), size (< 1 cm, ≥ 1 cm), and Yamada classification (type I, II, III, IV) of CRP were observed. The relationship between CRP and *H. pylori* infection was analyzed.

Clinical data of patients with CRP were collected, including age (≤ 50 years old, > 50 years old), gender (female, male), site of onset (left, right, left + right), the number of polyps (1, > 1), size (< 1 cm, ≥ 1 cm), the pathological type (non-adenomatous, adenomatous), and pedicle (with or without). The relationship between the p-S6K1 expression and the clinical as well as pathological characteristics of patients with CRP was analyzed.

Logistic regression model was used to explore the influencing factors of CRP, including age (≤ 50 years old, > 50 years old), gender (female, male), body mass index (BMI ≤ 23 kg/m^2^, > 24 kg/m^2^), *H. pylori* infection (negative, positive), smoking history (yes, no), hypertension history (yes, no), diabetes history (yes, no), apolipoprotein B (ApoB), lipoprotein (a) [Lipoprotein (a), Lp (a)] Apolipoprotein A1 (ApoA1), Apolipoprotein E (ApoE), etc.

### Statistical analysis

In this study, age, gender, pathological type, quantity, size, and location of onset were presented as [cases (%)] and compared using Chi-squared test. The measurement data of ApoB, Lp (a), ApoA1, ApoE, and so on were all determined for normality distribution and all fit the normal distribution. These measurement data were shown in the form of (‾*x* ± *s*). The measurement data between two groups were tested by *t*-test. The influencing factors of CRP were analyzed using multivariate logistic regression and covariance. Statistical data analysis was conducted using SPSS23.0 software in this study, and the difference was considered statistically significant with *P* < 0.05.

## Results

### The correlation between CRP and *H. pylori* Infection

There was significant difference in pathological type, site of onset, the number and size of polyps (*P* < 0.001, *P* = 0.037, *P* = 0.042 and *P* = 0.039) between the *H. pylori* negative group and the *H. pylori* positive group, but not in Yamada classification (*P* = 0.244, Table 1).

**Table 1:** The correlation between CRP and *H. pylori* infection [cases (%)]

Groups	The negative group (n = 52)	The positive group (n = 89)	*χ* ^*2*^	*P*
Pathological type (%)			26.089	**< 0.001**
Non-adenomatous	22 (78.57)	6 (26.55)		
Adenomatous	30 (21.43)	83 (73.45)		
Site of onset (%)			6.607	**0.037**
Left	18 (34.62)	18 (20.22)		
Right	27 (46.15)	37 (41.57)		
Left + Right	10 (19.23)	34 (38.20)		
Number of polyps			4.116	**0.042**
1	24 (46.15)	26 (29.21)		
≥ 2	28 (53.85)	63 (70.79)		
Size of polyps (cm)			4.265	**0.039**
< 1	47 (90.38)	68 (76.40)		
≥ 1	5 (9.62)	21 (23.60)		
Yamada classification (%)			4.167	0.244
Type I	14 (26.92)	12 (13.48)		
Type II	27 (51.92)	52 (58.43)		
Type III	5 (9.62)	10 (11.24)		
Type IV	6 (11.54)	15 (16.85)		

### The expression of p-S6K1 in CRP tissue

**Fig. 2:**
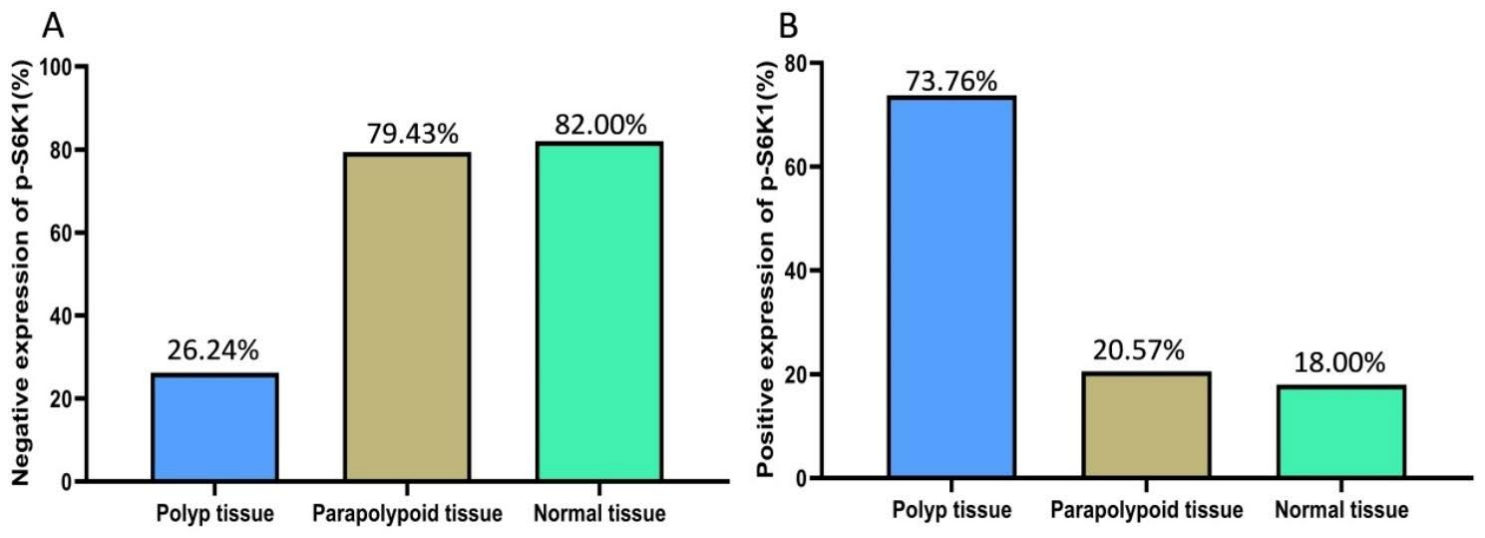
**Expression of p-S6K1 in CRP tissue and normal tissue A**: Negative expression rate of p-S6K1 in different tissues; **B**: Positive expression rate of p-S6K1 in different tissues

**Fig. 3:**
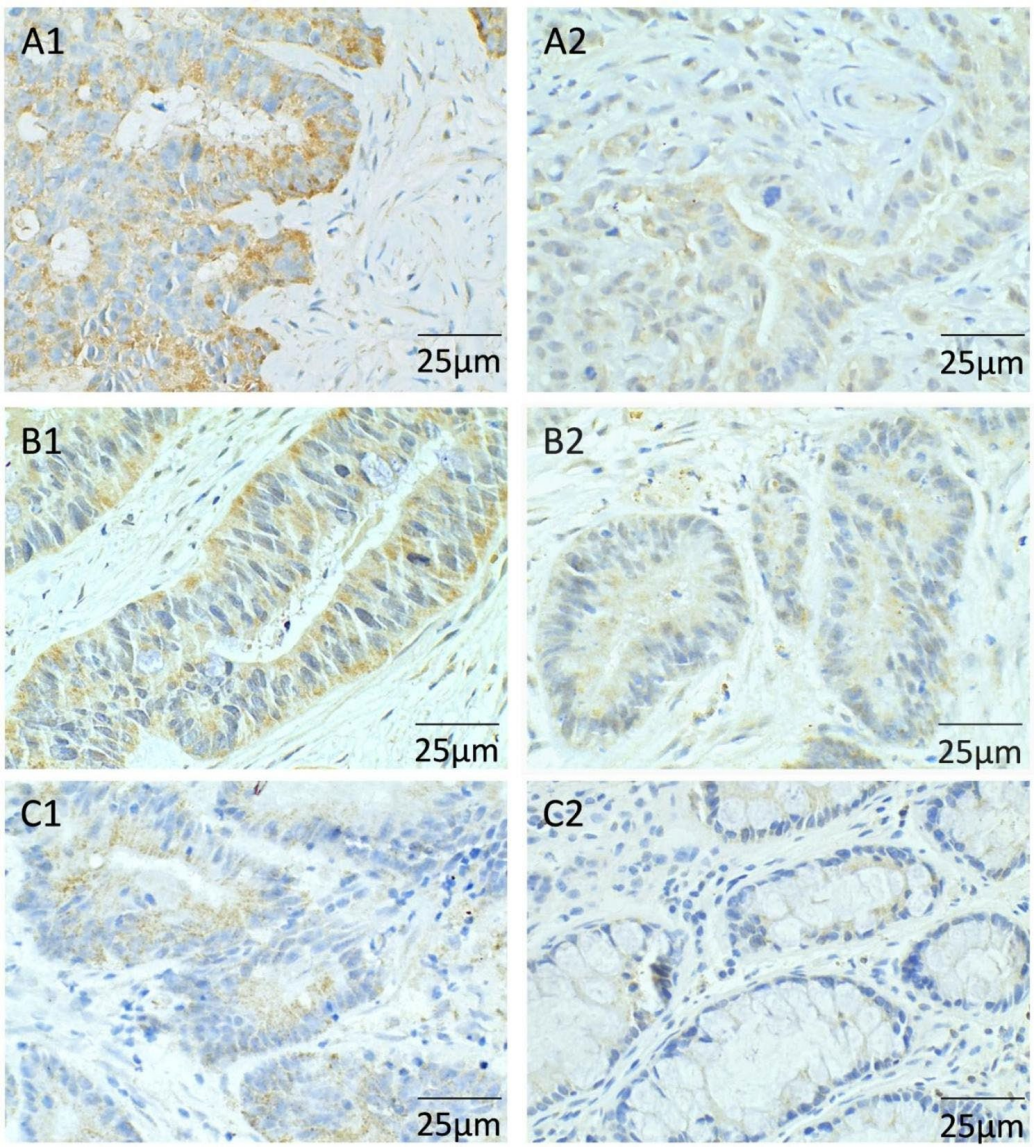
**The expression of p-S6K1 in CRP tissue detected by IHC A1**: Positive expression of p-S6K1 in polyp tissue; **A2**: Negative expression of p-S6K1 in polyp tissue **B1**: Positive expression of p-S6K1 in para-polyp tissue; **B2**: Negative expression of p-S6K1 in para-polyp tissue **C1**: Positive expression of p-S6K1 in normal intestinal tissue; **C2**: Negative expression of p-S6K1 in normal intestinal tissue

The percentage of the p-S6K1 positive expression in polyp tissues was higher than that in normal tissues and para-polyp tissues (*P* < 0.001). The expression of p-S6K1 in para-polyp tissues was higher than that in normal tissues, but without statistically significant difference (*P* = 0.696, Table 2; Figs. 2 and 3).

**Table 2:** The expression in CRP tissue [cases (%)]

Groups	Cases	Negative expression	Positive expression
Polyp tissue	141	37 (26.24)	104 (73.76)
Para-polyp tissue	141	112 (79.43)	29 (20.57)
Normal tissue	50	41 (82.00)	9 (18.00)
*χ* ^*2*^		96.247	
*P*		< 0.001	

### The relationship between p-S6K1 expression and clinical pathological characteristics in CRP patients

The p-S6K1 negative group showed statistically significant difference in number and pathological type of polyps, and the presence or absence of a pedicle compare to the p-S6K1 positive group (*P* = 0.006, *P <* 0.001 and *P* = 0.012). There was no statistically significant difference in age, gender, location of onset and size between two groups (*P* = 0.682, *P* = 0.354, *P* = 0.584 and *P* = 0.059, Table 3).

**Table 3:** The relationship between p-S6K1 expression and clinical pathological characteristics in CRP patients [cases (%)]

Groups	p-S6K1 positive group (n = 104)	p-S6K1 negative group (n = 37)	*χ* ^*2*^	*P*
Age (year)			0.168	0.682
≤ 50	30 (28.85)	12 (32.43)		
> 50	74 (71.15)	25 (67.57)		
Gender (%)			0.859	0.354
Female	47 (45.19)	20 (54.05)		
Male	57 (54.81)	17 (45.95)		
Site of onset (%)			1.077	0.584
Left	27 (25.96)	9 (24.32)		
Right	47 (45.19)	14 (37.84)		
Left + Right	30 (28.85)	14 (37.84)		
Number of polyps (number)			7.577	**0.006**
1	30 (28.85)	20 (54.05)		
≥ 2	74 (71.15)	17 (45.95)		
Size of polyps (cm)			3.560	0.059
< 1	81 (77.88)	34 (22.12)		
≥ 1	23 (91.89)	3 (8.11)		
Pathological type (%)			56.205	**< 0.001**
Non-adenomatous	8 (28.571)	20 (71.43)		
Adenomatous	96 (93.20)	7 (6.80)		
Pedicle			6.356	**0.012**
Yes	32 (30.77)	20 (54.05)		
No	72 (69.23)	17 (45.95)		

### Univariate analysis of the occurrence of CRP

Compared to the control, patients in the study group had higher proportion of BMI > 24 kg/m^2^, positive *H. pylori* infection and smoking history, and had much higher contents of ApoB and Lp(a) (*P* = 0.046, *P* = 0.001, *P* = 0.010, *P* < 0.001 and *P* < 0.001). There was no significant difference in age, gender, hypertension history, diabetes history, and the contents of ApoA1and ApoE between the two groups (*P* = 0.285, *P* = 0.853, *P* = 0.190, *P* = 0.606, *P* = 0.641 and *P* = 0.422, Table 4).

**Table 4:** Univariate analysis of the occurrence of CRP [cases (%), $(\bar x \pm s)$]

Groups	The study group (n = 141)	The control group (n = 50)	*t/χ* ^*2*^	*P*
Age (year)			1.145	0.285
≤ 50	42 (68.85)	19 (31.15)		
> 50	99 (76.15)	31 (23.85)		
Gender (%)			0.034	0.853
Female	67 (74.44)	23 (25.56)		
Male	74 (73.27)	27 (26.73)		
BMI (kg/m^2^)			3.974	**0.046**
≤ 23	70 (67.96)	33 (32.04)		
> 24	71 (80.68)	17 (19.32)		
*H. pylori* infection (%)			11.019	**0.001**
negative	52 (61.90)	32 (38.10)		
positive	89 (83.18)	18 (16.82)		
Smoking history (%)			6.621	**0.010**
Yes	43 (87.76)	6 (12.24)		
No	98 (69.01)	44 (30.99)		
Hypertension history (%)			1.717	0.190
Yes	32 (82.05)	7 (17.95)		
No	109 (71.71)	43 (28.29)		
Diabetes history (%)			0.267	0.606
Yes	18 (78.26)	5 (21.74)		
No	123 (73.21)	45 (26.79)		
ApoB (g/L)	1.12 ± 0.25	0.76 ± 0.14	9.649	**< 0.001**
ApoA1 (g/L)	1.10 ± 0.24	1.12 ± 0.31	0.467	0.641
ApoE (mg/L)	37.54 ± 8.46	38.72 ± 10.09	0.805	0.422
Lp(a) (mg/L)	135.79 ± 43.57	80.46 ± 40.16	7.870	**< 0.001**

### Logistic multifactor analysis affecting the occurrence of CRP

Logistic multifactor analysis showed that BMI, *H. pylori* infection, smoking history, ApoB, Lp(a) and the p-S6K1 positive expression were all risk factors for the development of CRP (*P* = 0.025, *P* = 0.020, *P* = 0.010, *P* = 0.005, *P* = 0.043 and *P* < 0.001, Table 5). Taking into account the interaction between the respective variables and other influencing factors of CRP, the covariance analysis was carried out with the respective variables as covariates, and the results were consistent with the logistic multivariate analysis (all *P* < 0.05).

**Table 5:** Logistic multifactor analysis affecting the occurrence of CRP

Groups	*B* value	Standard error	*Wald* value	*P* value	OR	95%*CI*	
						Lower limit	Upper limit
BMI	0.888	0.260	8.370	0.025	2.063	1.172	4.264
* H. pylori* infection	1.282	0.416	9.221	0.020	2.578	1.149	8.628
Smoking history	1.357	0.252	10.473	0.010	3.103	1.520	5.293
ApoB	1.346	0.421	12.335	0.005	4.169	1.517	10.939
Lp(a)	0.672	0.236	7.274	0.043	2.117	1.011	4.184
p-S6K1 positive expression	1.261	0.314	15.332	< 0.001	6.584	2.358	13.390

### 3 Discussion

CRP are abnormal protrusions on the surface of the large intestine, which are a common digestive system tumor in clinical practice. Colorectal adenomatous polyps are precancerous lesions and typically develop into CRC in the order of adenomatous, atypical hyperplasia, and cancer [11]. *H. pylori* is a common Gram-negative bacterium that parasitizes the gastric mucosa and secretes genes related to urease, vacuolar toxins, and cytotoxicity. Research has shown that the infection rate of *H. pylori* in the general population exceeds 50%, which makes it as the culprit of chronic gastritis, gastric ulcer, and gastric cancer [12]. By analyzing primary CRC patients and CRP patients who undergo colonoscopy and pathological diagnosis, the study has found that the incidence of *H. pylori* infection and the associated atrophic gastritis or intestinal metaplasia largely increase the risk of CRP and CRC [13]. Another study found a significant positive correlation between *H. pylori* infection and the risk of CRC [14], whose results were similar to those in the present study that there were significant differences between the *H. pylori* negative group and the *H. pylori* positive group in terms of pathological type, site of onset, number and size. These above results indicated a close relationship between *H. pylori* infection and CRP. It is hypothesized that *H. pylori* infection increases the risk of CRP, and the reasons for the differences in study results may be as follows. First of all, due to the different criteria for the selection population, this study screened the study group and the control group from the physical examination population. Secondly, it may be that the method of defining *H. pylori* infection is different. This study uses 13 C breath test to determine whether there is *H. pylori* infection, whiel some studies are by detecting serum anti-*H. pylori* infection antibodies, and some studies are detected by immunohistochemistry. Thus, different detection methods may result in different *H. pylori* infection rates, and statistical results are not the same. To explore the possible causes of the relationship between *H. pylori* infection and CRP, *H. pylori* infection up-regulates the expression of matrix metalloproteinases, which is not only involved in the occurrence of CRC with adenomatous polyps, but also may be involved in the initiation of invasion and metastasis cascade of CRC. In addition, *H. pylori* infection induces the abnormality of auxin secreted by gastric mucosa, and auxin can further prevent the occurrence of adenomatous polyps and malignant tumors [15, 16]. Therefore, treatment in *H. pylori* infection may help prevent the occurrence of CRP.

S6K1 is a key regulator for protein translation, which can be activated by mTOR and phosphorylated, promoting the translation of mRNA, and then expressing proteins related to the growth and proliferation of a large number of cells in the body [17]. Research has found [7] that the mTOR-S6K1 pathway is closely related to the proliferation and migration of cancer cells. There is a closely regulatory relationship between fibroblast growth factor 1 (FGF1) and the mTOR-S6K1 pathway, which may promote tumor cell proliferation and metastasis by regulating the AKT mTOR-S6K1 signaling pathway in various tumors. It has also been found that the expression level of GL1/p-S6K1 in colon cell carcinoma tissues was significantly related to lymph node metastasis and TNM stage, the survival time of patients with positive expression of GL1/p-S6K1 was shorter, and the overall survival rate of patients was worse than that of patients with single positive expression [18]. In addition, GLI1 and p-S6K1 expression are thought to be independent factors affecting patient outcomes. According to previous reports [6], the expression of p-S6K1 was significantly elevated in CRC tissue, and the expression level of inflammatory NLRP3 is positively correlated with the expression level of p-S6K1, which suggest that p-S6K1 may work together with inflammatory factors to induce tumor-initiating inflammation or stimulate cytokines by secreting growth or promoting tumor growth by mediating T cell inhibition. The results of this study showed that compared with normal tissues and parapolyp tissues, the proportion of positive expression of p-S6K1 in polyp tissues increased significantly, the expression level of p-S6K1 increased significantly, and its expression was significantly related to the number, pathological type and pedicle of CRP. Therefore, this study concluded that p-S6K1 played an important role in the formation of CRP, and detecting its expression in colonic lesion tissues could help determine the disease progression of CRP patients. Taken together, the analysis of positive expression of p-S6K1 may provide a basis for the diagnosis and development of the patient.

In addition, the results of this study found that body mass index, *H. pylori* infection, smoking history, positive expression of ApoB, Lp (a), and p-S6K1 were all risk factors affecting the occurrence of CRP. To explore the causes, the occurrence and development of CRP are an important process, and its occurrence may be closely related to metabolic abnormalities (BMI, blood sugar, blood lipids), hyperinsulinemia, and so on. The study has found [19] that in populations with lower obesity rates, the association between BMI and colorectal adenomas is inversely J-shaped, and underweight is closely associated with an increase in patient morbidity. *H. pylori* infection is the main risk factor for colorectal adenomatous polyps [15]. Smoking is a well-known modifiable risk factor for CRP. Previous study has found [20] that frequent smoking often leads to the occurrence of individual polyps, small polyps, rectal polyps, and so on, and the number and duration of daily smoking are also closely related to the risk of CRP. Both smoking cessation and obesity management may reduce the risk of CRP [21]. ApoB and Lp (a) are indicators that reflect blood lipid levels. Research has found [22, 23] that high levels of ApoB and the number of CRP are risk factors for the occurrence and development of CRP, and Lp (a) plays an important role in the early diagnosis of CRC. To explore the causes, long term lipid stimulation can inhibit the anti-tumor immune response, promote the occurrence and distant metastasis of CRC, and inhibit the function of immune infiltrating cells in the tumor microenvironment, thus accelerating tumor progression. As a potential early-stage lesion of CRC, more attention should be paid to the risk factors of CRP. Besides, better prevention and management of CRP is also needed [24]. This study discussed the various pathogenesis of CRP, and also found that the pathogenic process was not caused by a single factor, but was the result of the interaction and interaction of many factors. The study further confirmed the potential role of p-S6K1 positive expression in the pathogenesis of CRP, which might be an indicator of the severity of intestinal polyposis and whether it would develop to cancer in the future.

In general, *H. pylori* infection was closely related to the pathological type, site of onset, the number and size of CRP. p-S6K1 was highly expressed in CRP, and was positively related to the number, the pathological type and pedicle of polyps. *H. pylori* infection and positive p-S6K1 expression were independent risk factors for CRP. To explore the association between *H. pylori* infection, as well as p-S6K1 and CRP, it is hoped that it will help to formulate a more rigorous colorectal cancer screening program for *H. pylori*-positive individuals, and at the same time find a new direction for the prevention of CRP and colorectal cancer, and provide some help for future research. Analyzing these indicators exerts an important role in determining the development of CRP and preventing diseases. However, there are certain limitations in this study. This study was a retrospective study, which failed to obtain enough histopathology reports of polyps from each subject. Thus, future research is expected to explore the risk factors of CRP of different pathological types, and explore the mechanism in the occurrence and development of CRP. In addition, 50 cases were included in the healthy control group in this study, and the independent variables were 6, thus, the number of samples at the level with the least number of dependent variables was 5–10 times the number of independent variables. Although the number of cases in this study has no effect on the results, due to the small sample size of this study, the preliminary results of the study have not been multiple-corrected. Thus, the possibility of false positives cannot be ruled out, and subsequent studies should increase the sample size and standardize the trial design to clarify the mechanism of p-S6K1 in affecting the pathogenesis and clinical outcome of CRP, so as to better apply it in clinical diagnosis and treatment.

## Data Availability

The datasets used and/or analyzed during the current study are available from the corresponding author on reasonable request.
